# Attispdella Reale Accademia Medica Di Roma, 1886-1887

**Published:** 1888-04

**Authors:** 


**Attispdella Reale Accademia Medica di Roma, 1886-1887.** (Transactions of the Royal Academy of Medicine of Rome.) Tipografia Fratelli Centenari, Roma, 1887; pp. 340, 4to.

In no other country of Europe is better or more thorough work being done for the advancement of medical science than in Italy. It is in the departments of microscopical anatomy and pathology especially that the Italians excel. The universities and the Royal Academy of Medicine being under the patronage of the crown, are able to do thorough work and publish results in excellent form. The present volume is a beautiful specimen of typographical work, illustrated by eighteen lithographic plates, many of them in colors. The following are the contents: (1) Brain and Skull of a Microcephalus, by G. Mingazzini and O. Ferraresi; (2) Catarrhal Pneumonia resulting from Pneumothorax, by Giuseppina Cattani; (3) Anthropology of Tierra del Fuego, by G. Sergi, Professor of Anthropology in the University of Rome; (4) On the Ossification of the Periotic Capsules in Man and other Mammals, by E. Ficalbi; (5) Anatomical Observations on the Crania of 75 Lunatics, by Giovanni Mingazzini; (6) Minute Anatomy of the Olivary Body in Man, by Livio Vincenzi; (7) On the Normal Development and some Alterations of the Human Skin, by Sebastiano Giovanni; (8) A Case of Glanders in Man, by O. Ferraresi and G. Guarnieri; (9) Immediate Union of Wounds of Stomach in relation to various forms of Suture, by A. Poggi; (10) Researches on some Cystocerci of the arcolar tissues, by C. Crety; (11) Investigations of the Alterations of the Liver in Malarial Diseases, by G. Guarnieri; (12) On Phlegmonous Gastritis, by O. Ferraresi; (13) On Malarial Infection, by E. Marchiafava and A. Celli; (14) The Vasomotors and Vasomotor Centers in the Spinal Cord and Brain; the Vasodilator Nerves in the Posterior Roots of the Cord, by Pietro Bonuzzi.

